# Paraneoplastic and Other Autoimmune Encephalitides: Antineuronal Antibodies, T Lymphocytes, and Questions of Pathogenesis

**DOI:** 10.3389/fneur.2021.744653

**Published:** 2022-01-17

**Authors:** John E. Greenlee, Noel G. Carlson, Justin R. Abbatemarco, Ida Herdlevær, Stacey L. Clardy, Christian A. Vedeler

**Affiliations:** ^1^Neurology Service, George E. Wahlen Veterans Affairs Health Care System, Salt Lake City, UT, United States; ^2^Department of Neurology, University of Utah, Salt Lake City, UT, United States; ^3^Geriatric Research, Education, and Clinical Center (GRECC), George E. Wahlen Veterans Affairs Health Care System, Salt Lake City, UT, United States; ^4^Department of Neurobiology, University of Utah, Salt Lake City, UT, United States; ^5^Mellen Center for Multiple Sclerosis Treatment and Research, Neurological Institute, Cleveland Clinic Foundation, Cleveland, OH, United States; ^6^Neuro-SysMed, Department of Neurology, Haukeland University Hospital, Bergen, Norway; ^7^Department of Clinical Medicine, University of Bergen, Bergen, Norway

**Keywords:** autoimmune neurology, autoimmune encephalitis, paraneoplastic neurological syndromes, tissue culture, animal models, immune checkpoint inhibitors, treatment

## Abstract

Autoimmune and paraneoplastic encephalitides represent an increasingly recognized cause of devastating human illness as well as an emerging area of neurological injury associated with immune checkpoint inhibitors. Two groups of antibodies have been detected in affected patients. Antibodies in the first group are directed against neuronal cell surface membrane proteins and are exemplified by antibodies directed against the N-methyl-D-aspartate receptor (anti-NMDAR), found in patients with autoimmune encephalitis, and antibodies directed against the leucine-rich glioma-inactivated 1 protein (anti-LGI1), associated with faciobrachial dystonic seizures and limbic encephalitis. Antibodies in this group produce non-lethal neuronal dysfunction, and their associated conditions often respond to treatment. Antibodies in the second group, as exemplified by anti-Yo antibody, found in patients with rapidly progressive cerebellar syndrome, and anti-Hu antibody, associated with encephalomyelitis, react with intracellular neuronal antigens. These antibodies are characteristically found in patients with underlying malignancy, and neurological impairment is the result of neuronal death. Within the last few years, major advances have been made in understanding the pathogenesis of neurological disorders associated with antibodies against neuronal cell surface antigens. In contrast, the events that lead to neuronal death in conditions associated with antibodies directed against intracellular antigens, such as anti-Yo and anti-Hu, remain poorly understood, and the respective roles of antibodies and T lymphocytes in causing neuronal injury have not been defined in an animal model. In this review, we discuss current knowledge of these two groups of antibodies in terms of their discovery, how they arise, the interaction of both types of antibodies with their molecular targets, and the attempts that have been made to reproduce human neuronal injury in tissue culture models and experimental animals. We then discuss the emerging area of autoimmune neuronal injury associated with immune checkpoint inhibitors and the implications of current research for the treatment of affected patients.

## Introduction

Autoimmune encephalitides represent a rapidly expanding—and increasingly important—group of disorders characterized by the presence of an immunoglobulin G (IgG) antibody response directed against neuronal proteins. Although these antibodies were initially identified in patients with paraneoplastic (non-metastatic) neurological complications of underlying systemic cancer, autoimmune encephalitides are significantly more common in patients without neoplasia (1, 2) and are also recognized in patients treated with immune checkpoint inhibitors.

Antibodies associated with autoimmune encephalitides fall into two groups that are divided according to antigenic target. The first group, frequently paraneoplastic, is directed against neuronal proteins located in the cytoplasm and/or nuclei. The second, and much larger, group is directed against receptors or other proteins located on the neuronal cell surface membrane. Affected patients in these two groups can differ by the presence or absence of underlying neoplasia, the mechanisms of neuronal injury, and their response to treatment (3–5). Although major advances have been made in understanding the pathogenesis of conditions associated with antibodies against neuronal cell surface membrane antigens, the pathogenesis of syndromes associated with antibodies to intracellular neuronal proteins remains poorly understood. In this review, we will contrast antibodies to cell surface membrane antigens to antibodies directed against intracellular neuronal antigens, in terms of their discovery, how these antibodies may arise, and the roles of antibody and T cell-mediated immune response in producing neuronal injury. We will then discuss the emerging area of syndromes of autoimmune neuronal injury associated with immune checkpoint inhibitors and the implications current research may have on the treatment of affected patients. Conditions associated with antibody responses to non-neuronal CNS antigens, such as anti-glial fibrillary acidic protein (GFAP), anti-aquaporin 4 (Aqp4), or myelin-associated glycoprotein (MOG) or to antigens unrelated to the nervous system are outside the scope of this review.

## Paraneoplastic Neurological Disease and the Discovery of Antineuronal Antibodies

The concept that patients with cancer could develop syndromes of neurological injury in the absence of tumor metastasis or direct spread—conditions that are now termed “paraneoplastic neurological syndromes”—was introduced by Oppenheim in 1888, who reported the occurrence of central nervous system symptoms in a patient with uterine cancer in whom no evidence of tumor metastasis in the brain could be found at autopsy (6, 7). Around the same time, in 1890, M. Auché described peripheral nervous system symptoms in cancer patients (8). Recognition that these disorders constitute a novel area of neurological disease and their categorization into specific neurological syndromes such as subacute cerebellar degeneration [previously also termed cortical cerebellar degeneration and recently renamed “rapidly progressive cerebellar syndrome” (9)], limbic encephalitis, and sensory neuronopathy came through the work of multiple individuals over the next 70 years (10, 11) (Table 1). Demonstration that these disorders might be accompanied by an autoantibody response was first made by Wilkinson and Zeromski, who identified antibodies binding to neuronal cytoplasm and nuclei in patients with cancer and sensory neuronopathy (12), and subsequently by Trotter et al., who found antibodies to cerebellar Purkinje cells in a patient with Hodgkin's disease and cerebellar ataxia (13). Definitive association of an antineuronal antibody response in paraneoplastic neurological disease began with a report by Greenlee and Brashear in 1983; they identified the antibody now known as “anti-Yo” (“PCA1”) in patients with cerebellar degeneration in the setting of ovarian cancer (14) and with subsequent confirmatory work by Jaeckle et al. in 1985 (15). Soon thereafter Graus et al. and Greenlee and Lipton identified what is now known as anti-Hu (“ANNA-1”) antibody in patients with sensory neuronopathy and cerebellar degeneration, respectively (16, 17). Over the ensuing years, multiple additional autoantibodies have been identified in patients with paraneoplastic neurological disease: almost all of these antibodies are directed against intracellular neuronal antigens.

**Table 1:** Paraneoplastic neurological disorders)^a^.

**Syndromes affecting the central nervous system**
Cortical cerebellar degeneration (Rapidly progressive cerebellar syndrome)^b^
Encephalomyelitis
Limbic encephalitis
Bulbar encephalitis
Cerebellar degeneration (encephalitis)^b^
Myelitis
Intractable status epilepticus
Opsoclonus/ataxia
Paraneoplastic stiff-person syndrome
**Syndromes affecting ganglionic neurons**
Dorsal sensory neuronopathy
Autonomic neuronopathy (manifested as orthostatic hypotension, gastroparesis, etc.)
**Syndromes affecting the myoneural junction**
Lambert-Eaton myasthenic syndrome in the setting of small cell cancer
Myasthenia gravis in the setting of thymoma
**Syndromes affecting peripheral nerves**
Sensorimotor neuropathy
Axonal neuropathy
Mononeuritis multiplex: paraneoplastic vasculitis of peripheral nerves

a *Reprinted from Greenlee, Current Treatment Options in Neurology, 2010 (3)*.

b *Graus et al., In the most recent updated criteria for paraneoplastic neurologic syndromes have renamed cerebellar degeneration as “rapidly progressive cerebellar syndrome (9)”*.

The discovery of a second group of antineuronal autoantibodies, reactive against neuronal surface proteins, opened up a window into an entirely new category of neurological disease. Early work identified antibodies directed against the metabotropic glutamate receptor, mGluR1, in rare patients with Hodgkin's disease and cerebellar ataxia (18) and of antibodies directed against mGluR5 in patients with limbic encephalitis associated with Hodgkin's disease (19). Major advances, however, came through three important discoveries: (1) the detection of antibodies against components of the voltage-gated potassium channel (VGKC) complex in patients with Morvan's syndrome (20); (2) the subsequent association of this group of antibodies with limbic encephalitis and the syndrome of faciobrachial dystonic seizures (21–23); and (3) the identification of antibodies to the N-methyl-D-aspartate receptor (NMDAR) in young women with ovarian teratomas and encephalitis, with the subsequent recognition that an identical neurological syndrome associated with anti-NMDAR antibodies could occur in the absence of neoplasia (24, 25). Subsequent studies of patients with Morvan's syndrome or with faciobrachial dystonic seizures showed that the antibodies in both conditions are not directed against VGKC *per se* but rather to the adjacent surface membrane protein, contactin-associated protein-like 2 (Caspr2), and the channel accessory protein, LGI1, respectively (26). Many additional autoantibodies directed against neuronal membrane antigens have since been associated with neurological disease. It is now recognized that the overall burden of neurological disease associated with these antibodies not only outweighs that associated with antibodies directed against intraneuronal proteins but is also more common overall than viral encephalitides (1). Terminology for autoimmune neurological conditions associated with neoplasia has recently been revised, and the risk of neoplasia for different anti-neuronal and other antibodies has been stratified (9).

## Antibodies to Neuronal Surface Membrane Proteins

Antineuronal antibodies against neuronal membrane proteins represent the most commonly detected antibodies associated with autoimmune encephalitis, with a steadily growing list of over 50 different autoantibodies related to human neurological disease (Table 2). Virtually all of these antibodies are directed against known membrane proteins and may target neurotransmitter receptors. These include NMDAR, 2-amino-3-(5-methyl-3-oxo-1,2-oxazol-4-yl) propanoic acid receptors (AMPAR), γ-aminobutyric acid (GABA)_A_R or GABA_B_R, metabotropic glutamate receptors, ion channel complexes including P/Q voltage-gated calcium channels, as well as other membrane sites associated with neuronal growth or differentiation, such as immunoglobulin-like cell adhesion molecule (IGLON5). Although the syndromes associated with many of these antibodies are often categorized as “limbic encephalitis,” their semiology is more complex and may involve not only hippocampus and associated limbic structures, but also the brainstem, cerebellum, and, less frequently, spinal cord. The most common of these antibodies is anti-NMDAR. In the California Encephalitis Project, anti-NMDAR encephalitis was identified over four times more frequently than encephalitides due to herpes simplex type 1 virus, varicella-zoster virus, or West Nile virus (2, 27).

**Table 2:** Representative antibodies against synaptic or other neuronal cell surface proteins and their associated clinical syndromes.

**Anti-body**	**Major clinical syndromes**	**Major associated neoplasms**
AntiAMPAR	Limbic encephalitis	Small cell lung carcinoma Breast carcinoma Thymoma
Anti-Caspr2	Limbic encephalitis Morvan's syndrome	Tumor associations uncommon (Thymoma)
Anti-DPPX	Encephalopathy Myelopathy GI dysmotility	Tumor associations uncommon (Lymphoma)
Anti-DR2	Parkinsonism Encephalitis	No tumor association reported
Anti-GABA_A_R	Encephalitis Epilepsy	Tumor association uncommon (Thymoma, Hodgkin's disease, multiple myeloma)
Anti-GABA_B_R	Epilepsy Limbic encephalitis Opsoclonus myoclonus	Small cell lung cancer
Anti-Glycine receptor	PERM Stiff Person Spectrum Disorder	Tumor associations uncommon
Anti-IGLON5	Dementia Sleep disorder Respiratory impairment	(Thymoma)
Anti-NMDAR	Limbic encephalitis Psychosis Epilepsy Movement disorders Psychosis Catatonia	Ovarian or testicular teratoma
Anti-mGluR1	Cerebellar ataxia	Hodgkin's disease
Anti-mGluR5	Limbic encephalitis “Ophelia syndrome”	Hodgkin's disease
Anti-P/Q type VGCC	Cerebellar ataxia (Lambert-Eaton myasthenic syndrome)	Small cell lung cancer

*AMPAR, 2-amino-3-(5-methyl-3-oxo-1,2-oxazol-4-yl) propanoic acid receptor; Caspr2, contactin-associated protein-like 2; DPPX, dipeptidyl-peptidase-like protein 6 encephalitis; D2R, dopamine 2 receptor; GABA, γ-aminobutyric acid; LGI1, Leucine-rich glioma-inactivated NMDA-R, anti-N-methyl D-aspartate receptor encephalitis; mGluR1, metabotropic glutamate receptor 1; VGCC, voltage-gated calcium channel*.

## Antibodies to Intracellular Neuronal Proteins

Antibodies against intracellular neuronal proteins and their associated diseases are significantly less common than those associated with antibodies directed against cell surface membrane antigens (Table 3), and their role in disease pathogenesis, vs. that of T lymphocytes, is not known. Some, but not all, of these antibodies are directed against known intracellular proteins: these include anti-GAD65, anti-amphiphysin, and antibodies to collapsin response-mediator protein (CRMP5). Other antibodies, however, such as anti-Yo, anti-Hu, anti-Ri, and antibodies of the anti-Ma group, target intracellular antigens whose specific biological functions have not been fully elucidated. Anti-Yo antibodies have been reported to bind to the rough endoplasmic reticulum and Golgi apparatus within Purkinje cells (28, 29), and cloning of the Yo antigen has identified two closely related proteins, a 62 kDa protein, CDR2 (30), and a closely related 53 kDa protein, CDR2L (31). Both proteins contain a leucine zipper motif. However, antibodies to CDR2L more closely duplicate the antibody binding characteristics of native anti-Yo antibodies. In addition, CDR2L reacts with ribosomal proteins, similar to the ultrastructural localization of the Yo antigen seen with intact human anti-Yo antibodies, whereas CDR2 labels nuclear speckle proteins (28, 29, 31, 32). Studies by de Graaff et al. using mass spectrometry and transfected HLA cells, indicated that 12/12 anti-Tr sera, associated with cerebellar degeneration in the setting of Hodgkin's disease, reacted with glycosylated forms of the transmembrane Delta/Notch-like epidermal growth factor-related receptor (DNER) (33). Earlier studies by Graus et al., using confocal and immune electron microscopy, detected anti-Tr immunolabelling of Purkinje cell cytosol, the endoplasmic reticulum, dendrites, and the outer surface of the endoplasmic reticulum of neurons in the molecular layer, consistent with the transmembrane/intracellular distribution of DNER in Purkinje and related neurons (34).

**Table 3:** Representative antibodies against intracellular neuronal proteins and their associated clinical syndromes.

**Antibody**	**Major central nervous system syndromes**	**Major associated neoplasms**
**Antibodies reacting with cytoplasmic and/or nuclear antigens**		
Anti-Yo (PCA1)	Subacute cerebellar degeneration [*Recently renamed “Rapidly progressive cerebellar syndrome”* (9)]	Carcinoma of the ovary, uterus, or fallopian tube; carcinoma of the breast
Anti-Hu (ANNA1)	Encephalomyelitis Subacute cerebellar degeneration Sensory neuronopathy Autonomic failure	Small cell lung carcinoma (Myxoid chondrosarcoma) (Merkel cell and other neuroendocrine tumors)
Anti-Ri (ANNA2)	Opsoclonus-ataxia syndrome Cerebella ataxia Encephalomyelitis	Breast carcinoma Small cell lung cancer
Anti-ANNA3	Limbic encephalitis Encephalomyelitis Progressive cerebellar syndrome	Small cell lung cancer
Anti-CRMP5	Encephalomyelitis Progressive cerebellar syndrome Chorea	Small cell lung cancer Non-small cell lung cancer Thymoma
Anti-Kelch-like protein 11	Brainstem and cerebellar syndromes Cerebellar ataxia	Ovarian, testicular, or other teratomas Seminomas
Anti-Ma 1 & 2	Limbic encephalitis, Brainstem encephalitis Progressive cerebellar syndrome	Ma1: Small cell lung carcinoma Ma2: testicular seminoma
Anti-SOX1	Progressive cerebellar syndrome	Small cell lung cancer (Non-small cell lung cancer)
Anti-Tr^a^	Subacute cerebellar degeneration	Hodgkin's disease
**Antibodies reactive with intracellular synaptic or other**		
**membrane antigens**		
Anti-Amphiphysin	Stiff person syndrome Limbic encephalitis	Breast cancer Small cell lung cancer
Anti-GAD65	Stiff Person Spectrum Disorder Limbic Encephalitis Cerebellar ataxia	Tumor association rare (Multiple tumor types reported in individual patients: breast, lung, thymoma, other)

*ANNA, antineuronal nuclear antibody; SCLC, small cell lung carcinoma; NSCLC, non- small-cell lung cancer; CRMP-5, collapsin response mediator protein 5; GAD, Glutamic acid decarboxylase; KLHL11, Kelch-like protein-11; PCA, Purkinje cell cytoplasmic antigen; DNER, delta/notch-like epidermal growth factor-related receptor*.

a *Anti-Tr has been shown to react with glycosylated forms of the transmembrane delta/notch-like epidermal growth factor-related receptor (DNER). This protein is expressed intracellularly as well as at the neuronal cell membrane, and studies employing confocal and immune electron microscopy demonstrated anti-Tr immunolabelling of Purkinje cell cytosol, endoplasmic reticulum, dendrites as well as outer surface of the endoplasmic reticulum of neurons in the molecular layer (33, 34)*.

Antigens recognized by anti-Hu and anti-Ri antibodies are directed against RNA processing proteins encoded by the Hu and Nova family of genes, respectively (35–37). Both gene families have been associated with diverse biological functions. Anti-Hu antibody recognizes a family of genes, HuA, HuB, HuC, and HuD, which have been shown to play roles in RNA alternative splicing and polyadenylation; mRNA stability; shuttling of target mRNAs into the cytoplasm; and regulation of both localization and translation of transcripts within the cell cytoplasm and possibly also within neurites. Very little is known, however, about the actual alterations in neuronal function, which might occur following disruption of Hu protein function. One of the few studies addressing this question (35) demonstrated that Hu proteins regulate largely independent gene networks through control of overall transcript levels and alternative splicing. Importantly, these networks, despite their intrinsic diversity, intersect in controlling the synthesis of the major excitatory neurotransmitter, glutamate, and glutamate levels are severely compromised in Hu knockout mice (35). Given the importance of altered glutamate homeostasis in excitotoxicity, these data could provide a possible mechanism for anti-Hu-associated neuronal death.

In contrast to Hu proteins, expression of the Nova1 and Nova2 proteins recognized by anti-Ri antibodies is tightly restricted to post-mitotic neurons in the CNS, with the expression of Nova1 occurring predominantly in the brainstem and ventral spinal cord and Nova2 predominantly within the neocortex (38). Both proteins bind to RNA in a sequence-specific manner and regulate alternative splicing *in vitro*, and both appear to be involved in the maintenance of neuronal excitatory and inhibitory homeostasis (38, 39). Microarray analyses and work with Nova -/-knockout mice have demonstrated that the most Nova-regulated exons are located in genes encoding proteins with important synaptic functions: these may include N-type and P-type Ca_v_2 calcium channels as well as gephyrin, a protein that clusters inhibitory gamma-aminobutyric acid and glycine receptors (38–40).

As is the case with Hu proteins, however, no studies have addressed how the interaction of anti-Ri antibodies with their target antigens might alter RNA processing, nor have studies yet identified the downstream changes in RNA metabolism or protein encoding that might cause neuronal dysfunction or death. Beyond anti-Yo and anti-Hu, there are numerous other antibodies targeting intracellular proteins whose effects on neurons remain to be characterized, including the Ma antigens—recognized by anti-Ma and anti-Ta antibodies—that represent a family of proteins expressed in CNS neurons and in testis (41).

## Initiation of the Immune Response

The molecular events that lead to antineuronal antibody-associated neurological disease in patients without underlying neoplasia have not yet been identified. An exception to this is the occurrence of anti-NMDAR encephalitis as a late complication of herpes simplex encephalitis (42). The pathogenesis of this association has not been elucidated, but congenital deficiency of Toll-like receptor 3, a key protective factor against viral encephalitis, has been reported in patients in whom herpes simplex encephalitis was followed by anti-NMDAR encephalitis (43, 44).

In contrast, classical paraneoplastic syndromes, such as those associated with anti-Yo or anti-Hu antibodies, are unique among other types of systemic autoimmune disorders in that the antigenic stimuli that initiate the immune response have been identified and shown to be elicited by antigens expressed in patient tumors. The same is true for cases of anti-NMDAR encephalitis associated with ovarian teratomas. Expression of Yo antigen(s) in tumors found in patients with paraneoplastic cerebellar degeneration is well-documented, as is similar tumor expression of neuronal antigens in tumors of patients with anti-Hu, anti-Ri, anti-Ma2, and anti-NMDAR, and GABA_B_ antibody-associated encephalitides (41, 45–53). Similarly, GABA_B_ expression has been detected in thymoma biopsies in the setting of GABA_B_ encephalitis.

An important question has been whether expression of neuronal antigens is confined to the subset of tumors found in patients who experience paraneoplastic neuronal injury or whether these antigens are more commonly expressed by tumors but do not always cause neurologic injury, suggesting that additional host genetic or other factors may be involved in disease pathogenesis (54). Early work by Furneaux et al. suggested that anti-Yo antibodies labeled cells within ovarian carcinomas from those individuals with anti-Yo antibody-associated paraneoplastic cerebellar degeneration but did *not* label tumors from control patients with similar malignancies (45). Subsequent work, however, has demonstrated that two of the antigens detected by anti-Yo antibodies, CDR2 and CDR2L, can be detected in cancer patients both *with* and *without* neurological disease (55, 56). Similarly, although ovarian teratomas from patients both with and without NMDAR encephalitis express the GluN1 subunit of NMDAR, NMDAR encephalitis occurs in only a subset of these patients (51, 52). Hu antigens have been detected in small cell tumors from patients *with* and *without* neurological disease (47), and low titers of anti-Hu antibody have been detected in sera from neurologically asymptomatic patients with small cell tumors (57). The risk of underlying neoplasia associated with different antineuronal antibodies has been recently summarized by Graus et al. (9) (Table 4).

**Table 4:** Risk of underlying neoplasia associated with detection of major antineuronal antibodies^a^^,^^b^.

**High risk (>** **70% association with cancer)**	
Hu	Ri
CV2/CRMP5	Yo
SOX1	Ma2/Ma
PCA2 (MAP18)	Tr
Amphiphysin	KLHL 11
**Medium risk (30–70% association with cancer)**	
AMPAR (>50%)	P/Q VGCC (50/90%^c^)
GABA_B_R (>50%)	CASPR2 (50%)
mGluR5 (~50%)	NMDAR (38%)
**Low Risk (<30% association with cancer)**	
mGluR1 (30%)	
GABA_A_R (<30%)	DPPX (<10%)
CASPR2 (<30%)	GlyR (<10%)
GAD65 (<15%)	
LGI1 (<10%)	

a *Modified from Graus et al.: Updated Diagnostic Criteria for Paraneoplastic Neurologic Syndromes, Annals of Neurology 2021 (9)*.

b *Graus et al. also included risk of cancer associated with three antibodies to non-neuronal proteins: glial fibrillary acid protein (GFAP, AQP4, and MOG). These are not included in the table*.

c *Risk of associated cancer (small cell carcinoma) is 50% when the association is with LEMS but 90% if associated with rapidly progressive cerebellar syndrome*.

Work within the past few years has shed important light on the genomic and histopathological factors that separate patients with neoplasms who develop autoimmune neurological disease from those patients with similar neoplasms who remain neurologically unaffected. Anti-LGI1 encephalitis, although not usually a paraneoplastic condition, has been shown in genome-wide association studies (GWAS) to be highly associated with 27 single-nucleotide polymorphisms (SNPs) in the HLA-II region (58). Chefdeville et al., in studies of teratomas from patients with and without anti-NMDAR encephalitis, found that teratomas from NMDAR patients more frequently contained neuroglial elements than did control tumors, and frequently contained robust T and B cell inflammatory infiltrates (52). A minority of tumors also contained elements resembling neuroglial tumors, a finding which is rare in ovarian tumors overall (52). Hillary et al., in a study of 43 cancer patients with cerebellar degeneration and anti-Yo antibodies described the existence of HLA allele association with anti-Yo mediated paraneoplastic cerebellar degeneration. These investigators noted that the association is complex, suggesting that multiple epitopes within Yo or other antigens may be involved (59). Vialatte de Pémille et. al. reported that CDR2L, but not CDR2, is enriched in ovarian cancers from patients with anti-Yo antibody response (60). In an important study, Small et al. examined ovarian tumors from patients exhibiting anti-Yo antibody response (anti-Yo PCD patients) as compared to antibody-negative controls. Tumors from patients with anti-Yo antibodies differed from controls in showing more abundant—and often massive—T- and B-cell infiltration. In some instances, these infiltrates were organized into tertiary lymphoid structures located near apoptotic tumor cells and harboring CDR2L protein deposits, a spatial association suggesting immune attack (54). In contrast to anti-Yo negative controls, 65% of anti-Yo PCD tumors presented one or more somatic mutations in genes encoding the Yo antigen, with a predominance of missense mutations, and 59% of anti-Yo PCD tumors showed recurrent gains of the CDR2L gene with tumor protein overexpression. In aggregate, these data were thought to indicate that genetic alterations in tumor cells could trigger immune tolerance breakdown, resulting in extensive tumor infiltration by T and B lymphocytes and initiation of autoimmune disease.

## Pathogenic Mechanisms in Diseases Associated With Antibodies to Neuronal Surface Membrane Antigens

**Figure 1:**
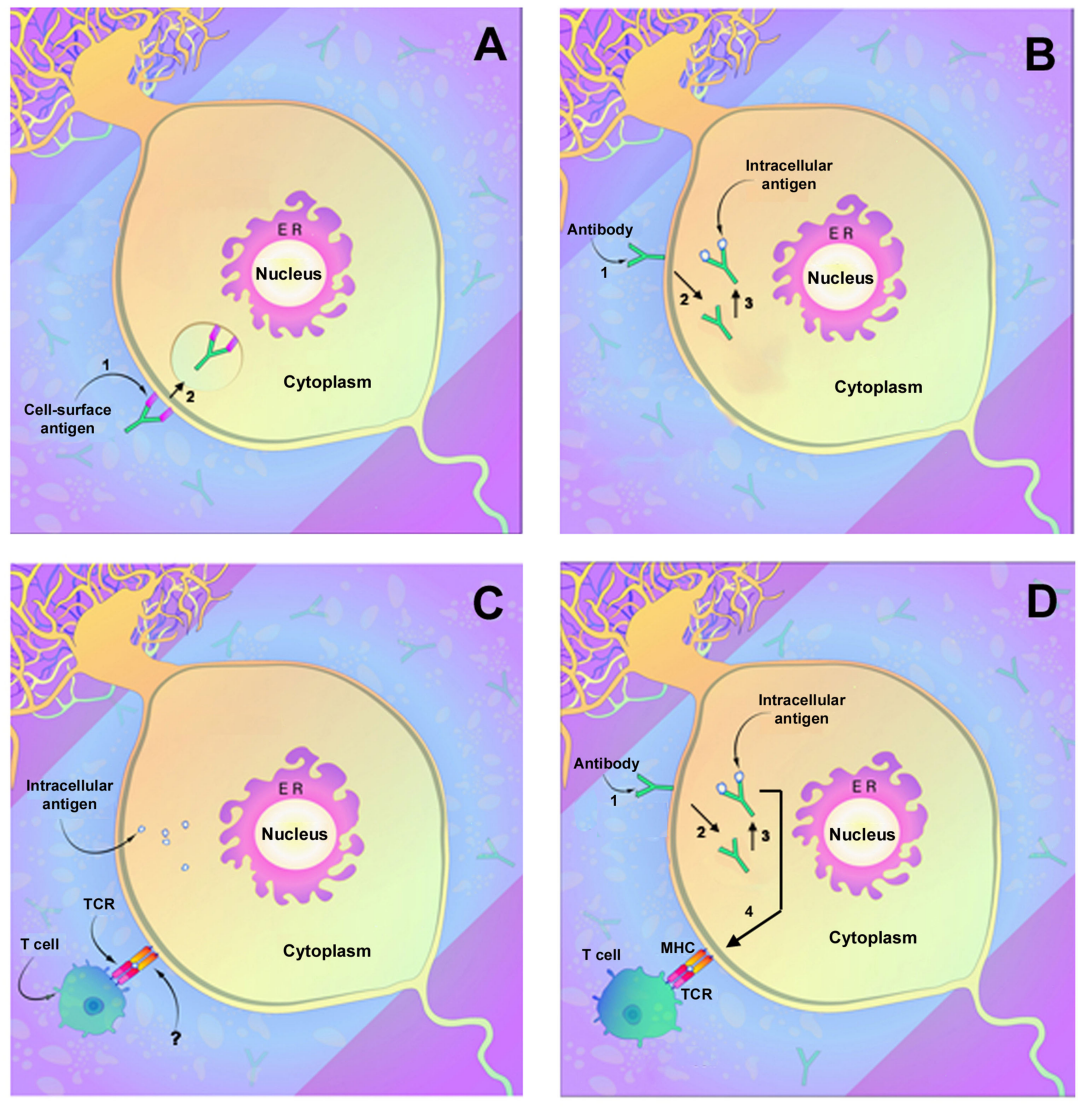
Demonstrated and potential mechanisms of autoimmune neuronal injury. **(A)** Immune attack directed against neuronal surface membrane antigens as has been shown to occur with antibodies such as anti-NMDAR. In this instance, the antibody can decrease receptor function through either (1) binding and inhibiting the receptor or (2) by cross-linking the receptors, which facilitate internalization of receptors and reduction in membrane receptor density. **(B)** Antibody uptake and antibody-mediated neuronal injury by antibodies directed against intracellular neuronal antigens such as anti-Yo or anti-Hu. (1) Antibody attaches to the neuronal membrane, possibly by Fc-related binding, and (2) is internalized. (3) Antibody binding to its intracellular target antigen results in neuronal injury or death. **(C)** Neuronal injury by T lymphocytes. Lymphocyte T cell receptors (TCRs) interact with target neurons and cause neuronal injury or death. An area of uncertainty is that mature neurons (as opposed to fetal neurons) do not express the MHC receptors normally required for T cell interaction, and the actual mechanism of neuronal recognition by T cells is undefined. **(D)** Possible two-step mechanism of immune attack directed against intracellular neuronal antigens. As in **(B)**, the antibody binds to the neuronal membrane (1) followed by internalization (2) and binding to target antigens with resultant neuronal injury (3). Injured neurons upregulate MHC receptors (4) allowing recognition by cytotoxic T lymphocytes that also contribute to cell death. [Modified from Herdlevaer et al. (32)].

Antibodies directed against neuronal membrane antigens play a direct role in disease pathogenesis. This has been most clearly demonstrated for antibodies directed against NMDAR and has been shown for several other anti-cell surface membrane antigens as well. Anti-NMDAR antibodies consistently target the N368/G369 region within the GluN1 subunit of NMDAR (61). *In vitro* studies of rat hippocampi demonstrate that antibody binding results in capping and cross-linking of receptor proteins, with subsequent receptor internalization and reduced presence of NMDAR clusters on the cell surface membrane (62) (Figure 1A). These studies parallel studies of receptor density in brains of rats infused intraventricularly with anti-NMDAR and also studies of receptor density in hippocampi obtained at autopsy from affected human patients (62). The binding of anti-NMDAR to its target receptor site did not appear to cause neuronal death, and reconstitution of synaptic density occurred when antibody titers were reduced (62). In all, these findings confirm a direct role for antibodies in the pathogenesis of anti-NMDAR encephalitis and, importantly, suggest that neuronal receptor function can recover and result in clinical improvement. Very few autopsy studies of anti-NMDAR encephalitis have been reported, and most of these have involved patients with a short duration of illness (63, 64). An as-yet unanswered question is thus whether prolonged neuronal exposure to anti-NMDAR antibodies in affected patients might eventually produce significant neuronal death or irreversible neuronal dysfunction.

Although antibodies to other neuronal surfaces and synaptic antigens have been less thoroughly studied, many produce similar effects on neurons. Anti-AMPAR antibodies have been shown to bind to the GluR1 and GluR2 subunits of AMPAR and to decrease receptor cluster density (65, 66), with alteration of inhibitory synaptic currents and vesicular c-aminobutyric acid transporter staining intensity (66). Antibodies to LGI1 bind to the ADAM23 (Disintegrin and metalloproteinase domain-containing protein 23) and ADAM22 within the trans-synaptic LGI1 complex and reduce total and synaptic levels of AMPA as well as of the voltage-gated potassium channel Kv1.1 (67). Antibodies to GABA_A_ receptors similarly cause a reduction in synaptic receptor clusters (68). In contrast, antibodies to Caspr2 interrupt the interaction between Caspr2 and contactin-2 without reducing membrane receptor density (69), and anti-Caspr2 antibodies, unlike many of the other antibodies to receptor proteins are of IgG subclass 4 (IgG4) (69). Although most autoantibodies directed against neuronal membrane antigens appear to affect only the function of the target antigen, anti-IGLON5 antibodies, often clinically associated with a neurodegenerative syndrome, decrease receptor cluster density followed by disorganization of the neuronal cytoskeleton (70). Similarly, antibodies to voltage-gated calcium channels, in addition to their effect on receptor *function*, can be internalized, resulting in neuronal death (71). Antibodies to amphiphysin, although a submembrane protein, behave much like antibodies to neuronal surface membrane antigens. Sommer et al., employing anti-amphiphysin antibodies, were successful in demonstrating not only neuronal antibody uptake but also dose-dependent stiffness and spasms mimicking human stiff person syndrome (72). Antibodies to GABA_A_ receptors similarly cause a reduction in synaptic receptor clusters (68).

## Pathogenic Mechanisms in Diseases Associated With Antibodies to Intracellular Neuronal Antigens

The discovery of paraneoplastic autoantibodies led to multiple attempts to produce an animal model of antibody-mediated paraneoplastic neurological injury using passive transfer of antibodies, immunization with recombinant antigen or relevant DNA sequences, or adoptive transfer of T lymphocytes (Table 5) (74–76, 78, 80). Although some of these studies documented neuronal antibody uptake, none have produced the neurological findings or the extensive neuronal destruction seen in human disease (Table 5). Based on the failure to produce neurological disease using antibodies, it has become widely thought that paraneoplastic neurological syndromes associated with antibodies such as anti-Yo or anti-Hu cannot be antibody-mediated and hence must be T-cell-mediated. In this concept, antibodies themselves are simply markers of underlying malignancy. Studies to induce neurological disease using T lymphocytes, which have also been unsuccessful, have received little attention (76, 83).

**Table 5:** Major experimental attempts to produce an animal model of paraneoplastic neurological disease associated with antibodies targeting intracellular neuronal antigens.

**References**	**Antigen targeted**	**Species**	**Method**	**Study duration**	**Outcome**
Graus et al. (73)	Yo	Guinea pigs	Intraventricular infusion of anti-Yo or normal IgG	15 days	Uptake of both anti-Yo and normal IgG by Purkinje cells on day 16 but not at days 22 and 45. No observed Purkinje cell death or neurological change in animals
Tanaka et al. (74)	Yo	Mice	Intracranial injection of human anti-Yo IgG with and without complement or activated monocytes	Up to 50 h	Uptake of human anti-Yo IgG by Purkinje cells. No detected Purkinje cell loss
		Mice	Immunization with recombinant Yo protein	15 days for pathology, 3 months for observation	Development of high antibody titers No definite uptake of antibody by Purkinje cell
		Rats	Intraventricular injection	1 week	No Purkinje cell loss. Brains not studied for antibody uptake by neurons.
Tanaka et al. (75)	Yo	Mice	Immunization of multiple mouse strains with recombinant Yo protein	>2 months	No Purkinje cell loss or ataxia Strong peripheral anti-Yo production Brains not studied for uptake by neurons.
Tanaka et al. (76)	Yo	Mice	Injection of human anti-Yo IgG into occipital lobes	50 h	Antibody uptake by Purkinje cells. No Purkinje cell loss
		Mice	Injection of mouse recombinant anti-Yo IgG into mouse brain parenchyma	3–4 months	High titers of anti-Yo antibody. No neurological abnormalities No Purkinje cell loss Brains not studied for antibody uptake by neurons
		Mice	Adoptive transfer) of lymphocytes from mice immunized with recombinant Yo protein with and without recombinant anti-Yo antibodies	1 month	No neurological abnormalities No Purkinje cell loss Brains not studied for antibody uptake by neurons.
		SCID Mice	Adoptive transfer of peripheral mononuclear cells from a patient with anti-Yo antibody	1 month	No neurological abnormalities No Purkinje cell loss Attempts to detect intraneuronal IgG not described
Greenlee et al. (77)	Yo	Rats	Intraperitoneal injection of human anti-Yo antibody following blood-brain barrier disruption	4 days	Anti-Yo IgG uptake by Purkinje cells. No evidence of Purkinje cell death No neurological abnormalities
Sillevis Smitt et al. (78)	Hu	Mice	Passive intravenous transfer of human anti-Hu IgG	48 h	No evidence of anti-Hu IgG in brains of animals perfused to remove intravascular IgG No evidence of antibody uptake by neurons in perfused brains)^a^
		Mice, Rats, Guinea pigs	Immunization with HuD recombinant protein	Up to 21 weeks	High serum antibody titers: No evidence of penetration of IgG into brain parenchyma or neurons in brains perfused to remove intravascular IgG
Tanaka et al. (79)	Yo	Mice	Immunization of female mice with recombinant protein; evaluation of offspring to detect transplacental passage of antibody to offspring with undeveloped blood-brain barriers	At birth and later	No Purkinje cell loss at birth No ataxia in newborn animals allowed to mature Brains not studied for antibody uptake.
Sakai et al. (80)	Yo	Mice	Immunization of mice with recombinant PCD17 protein generated using anti-Yo antibody (81)	1 year	Generation of high serum antibody titers. Presence of IgG in Purkinje cells of immunized mice, No identified Purkinje cell death or neurological abnormalities
Sakai et al. (82)	Yo	Mice	Immunization with DNA encoding recombinant PCD17 protein	Up to 1 year	Generation of antibody response which could lyse syngeneic myeloma cells pulsed with H-2K-restricted PCD17 peptide. No Purkinje cell loss or neurological abnormality Brains not studied for antibody uptake.
Pellkofer et al. (83)	Ma1		Adoptive transfer of lymphocytes from syngeneic rats immunized with recombinant Ma1 protein	9 days	Meningeal and perivascular inflammatory changes. No evidence of neuronal injury
Sakai et al. (84)	Yo	Mice	Immunization with recombinant yeast expressing recombinant (pcd17) Yo antigen	6 months	Generation of antibodies reactive with Purkinje cells and of T lymphocytes sensitized to pcd17 No clinical signs or Purkinje cell loss. Brains not studied for antibody uptake.

*This study contained important controls for the detection of adventitious entry of antibodies into neurons in post mortem tissue sections*.

### Current Knowledge of the Role of T Lymphocytes in Disease Pathogenesis

The presence of cytotoxic T lymphocytes in brains and CSF of patients with classical paraneoplastic neurological syndromes associated with anti-Yo, anti-Hu, and anti-Ma2 antibodies has been extensively documented (85–89). Cytotoxic (CD8^+^) lymphocytes have been demonstrated in the CSF of a patient with cerebellar degeneration and anti-Yo antibody response (90), and T cell clones recognizing the same antigen in brain and tumor tissue have been detected in the CSF of a patient with encephalomyelitis and anti-Hu antibodies (91). *However, not all patients with antibodies to intracellular neuronal antigens have evidence of an antigen-specific T cell response*: lymphocytic infiltrates have been absent in the brains of some patients with anti-Yo associated paraneoplastic cerebellar degeneration (14, 92); and some investigators have failed to detect cytotoxic T lymphocytes in serum or CSF of patients with anti-Hu antibodies and paraneoplastic neurological disease (93–95). An important, but unaddressed issue concerns the ability of cytotoxic T lymphocytes to target neurons since adult neurons lack the MHC class I or class II receptors normally required for recognition by T cells (Figure 1C). Recent studies by Yshii et al., however, documented upregulation of MHC class 1 molecule expression in Purkinje cells in an experimental model of paraneoplastic cerebellar injury following treatment with an immune checkpoint inhibitor (96), and in ongoing studies, we have observed similar neuronal upregulation of MHC class I receptors in slice cultures incubated with anti-Hu antibodies (Carlson et al., unpublished data). Taken together, these studies document the presence of autoreactive T cells in the brains of many affected patients and suggest a mechanism by which CD8^+^ T cells could recognize affected neurons. However, despite extensive attempts, no investigator has as yet developed an animal model of paraneoplastic neurological disease using T lymphocytes (Table 5) (76, 83). Attempts have included work by Tanaka et al. to produce neurological disease by adoptive transfer of mononuclear cells from a patient with paraneoplastic cerebellar degeneration into SCID (severe combined immunodeficiency disease**)** mice, attempts by the same group using passive transfer of T lymphocytes from mice immunized with Yo protein (76), and studies by Pellkofer et al., who studied the adoptive transfer of lymphocytes from animals immunized with the Ma-associated onconeural antigen, Pnma1, wherein recipient rats developed meningoencephalitis but not actual neuronal injury (83).

### Investigations Into the Role of Antibody

The role of antibodies reactive with intraneuronal antigens, such as anti-Yo or anti-Hu, in the pathogenesis of paraneoplastic neuronal injury has been a subject of controversy. In part this has been because cytotoxic T lymphocytes have been identified in the brains of affected patients, suggesting a T cell mechanism. The controversy also remains because attempts to produce disease using these antibodies in experimental animals have been unsuccessful. In addition, neurons have historically been thought to exclude IgG, and it has thus been thought that paraneoplastic antibodies such as anti-Yo or anti-Hu would be unable to enter living neurons and react with their target antigens (97, 98). Although this concept is widely stated, both *in vivo* and *in vitro* studies have demonstrated that IgG can enter neurons and that neuronal uptake of IgG can produce neuronal injury. Early work by Fabian et al. demonstrated entry of antibodies into the central nervous system in living animals (99, 100); and Griffin et al. have demonstrated neuronal uptake of IgG in mice infected with Sindbis virus (101). Graus et al. have shown Purkinje cell uptake of normal and anti-Yo IgG in guinea pigs following intraventricular infusion (73). In short-term experiments, Greenlee et al. demonstrated similar Purkinje cell uptake of anti-Yo antibodies following intraperitoneal injection of animals in the setting of blood–brain barrier disruption, and Tanaka observed similar neuronal uptake following intracranial injection (76, 77). Relevant to human disease, intraneuronal IgG has been found in autopsied brains of individuals with encephalitis associated with both anti-Hu and anti-amphiphysin antibodies (102–104).

The failure of previous attempts to produce an animal model of paraneoplastic neuronal injury could be due to a number of factors. First, no study to date has employed or generated antibodies proven to be cytotoxic to their target neurons *in vitro;* and failure to develop an animal model could thus reflect a failure to immunize animals with the correct antigen or to use the proper antibody in experiments involving passive transfer. Case in point are the attempts to produce Purkinje cell injury using the cloned Purkinje cell antigen, CDR2 as an antigen. It is now recognized that the major Yo antigen may be CDR2L rather than CDR2 and that antibodies directed against CDR2L most closely parallel the antigen-binding seen by human anti-Yo antibodies in cerebellar sections studied using immune electron microscopy as well as by immunohistochemistry and immunoprecipitation (28, 29, 31). It is thus possible that successful production of an animal model for anti-Yo antibody-associated cerebellar degeneration may require immunization with CDR2L rather than CDR2, or, given the possible roles of each antigen in Purkinje cell protein synthesis, that immunization with both proteins might be required (32). A second issue, involved in passive transfer experiments using human IgG, could be a failure of antibody-mediated pathogenicity to occur across species lines. In early work, Greenlee et al. demonstrated that there are differences in antibody reactivity of anti-Yo and anti-Hu antibodies among IgGs from different patients and also among different animal species (105). An additional challenge in developing an animal model is that detection of early, possibly widely scattered, neuronal loss could be difficult using the conventional histological methods employed in essentially all animal studies. Importantly, no study to date has used human paraneoplastic IgG to affinity purify target antigen(s) from neurons of the species to be studied and then employ these for direct immunization or to generate antibodies for passive transfer.

A final, major challenge in producing an animal model of human paraneoplastic disease using antibody has to do with achieving sustained exposure of neurons to antibodies across the blood-brain barrier, given that neurological symptoms in human cases associated with antibodies such as anti-Yo or anti-Hu are believed to be the result of *progressive* neuronal death over time. Robust neuronal uptake of IgG has been clearly demonstrated in three separate studies using direct intracranial injection, but antibody uptake has been minimal or has not occurred in longer-term immunization or passive transfer studies (73, 74, 77). As an example, Sillevis Smitt et al., in a carefully done study using passive transfer of human anti-Hu IgG, failed to show entry of IgG into brain parenchyma or neurons (78). Additional experiments employing immunization with recombinant HuD resulted in high antibody titers but, again, did not show antibody penetration across the blood-brain barrier or entry of antibodies into brain parenchyma or neurons within the timeframe of the study (78).

One approach to studying antibody-neuron interactions in the absence of a blood-brain barrier has been through the use of tissue culture systems. In older work, anti-Hu antibodies were shown to be taken up by rat cerebellar granule cells in dispersed cultures and to cause neuronal death (106). More recently, two groups of investigators have employed organotypic (slice) cultures of rodent brains to study the interaction of normal and paraneoplastic IgGs in the absence of a blood–brain barrier, using a system in which this interaction can be studied in real time. These studies have shown that, in slice culture, Purkinje and other neurons are able to take up and clear normal IgG (107). In contrast to normal IgG, internalized anti-Yo, anti-Hu, and anti-Ri IgGs *bind* to their target antigens, and accumulate intracellularly, with anti-Yo IgG concentrated predominantly in Purkinje cells and anti-Hu and anti-Ri antibodies in multiple neuronal populations (108–111) (Figure 1B). Antibody uptake is rapid and, in studies done in real time, can be observed within 4 h (109, 111). The effects of antibody accumulation differ among anti-Yo, anti-Hu, and anti-Ri antibodies. Anti-Yo causes cell death largely limited to Purkinje cells (111). In the anti-Yo experimental model, Terminal deoxynucleotidyl transferase dUTP nick end labeling (TUNEL) stains of injured Purkinje cells are negative, suggesting that cell death is non-apoptotic (111). In contrast, incubation of cultures with anti-Hu antibodies results in neuronal death which appears to be at least in part apoptotic (109). Although anti-Ri IgGs are widely taken up by neurons, neuronal death has *not* been detected. These observations raise questions as to whether anti-Ri antibody, at least initially, may cause neuronal dysfunction rather than neuronal death, in keeping with clinical observations showing partial clinical improvement in anti-Ri patients following treatment (109, 112). The actual mechanisms involved in neuronal death for both anti-Yo and anti-Hu antibodies are incompletely understood, and although multiple biological effects of anti-Hu and anti-Ri antibodies have been postulated, none has been directly associated with neuronal death or dysfunction. Of note, however, studies by Schubert et al. and Panja et al. indicate that anti-Yo antibodies may alter neuronal mitochondrial calcium homeostasis, possibly providing a mechanism for Purkinje cell death (110, 113). Studies addressing the functional consequences of uptake of anti-Ma2, anti-Tr, or other antibodies to intracellular neuronal proteins have not been reported.

Although *in vitro* studies with anti-Yo and anti-Hu antibodies suggest that paraneoplastic autoantibodies may play a direct role in neuronal injury in the absence of T lymphocytes, these findings have not been duplicated in living animals. The possibility also exists that antibody uptake could render neurons susceptible to attack by cytotoxic T cells by upregulation of neuronal MHC class I receptors or by other mechanisms (54, 96) (Figure 1D). Finally, it is possible that multiple of these mechanisms of pathogenicity may be at play during the course of the disease.

## Autoimmune and Paraneoplastic Encephalitides Associated With Immune Checkpoint Inhibitors

The advent of immune checkpoint inhibitors as treatments for advanced malignancy has been accompanied by the unintended induction of several categories of autoimmune neurological disease including meningoencephalitis, limbic encephalitis, polyradiculitis, cranial polyneuropathy, myasthenic syndrome, and myositis (114). In addition, the use of these agents has resulted in cases of disorders classically considered paraneoplastic, including sensory neuronopathy, limbic encephalitis, and cerebellar syndrome (115–120). The major antibodies detected have been anti-Hu, and anti-Ma2, although single cases have been associated with anti-Ri, anti-CRMP5, anti-PCA-2, anti-GAD65, and other antibodies (119, 121–126). To date, an association with anti-Yo antibodies has not been reported. Three cases have been described with complex antibody responses involving anti-Hu and other paraneoplastic autoantibodies including anti-CRMP5/CV2, anti-SOX-1, anti-VGKC, or anti-NMDAR (115, 127). Cases associated with anti-Hu antibody response have occurred predominantly in patients with small cell or Merkel cell tumors, or patients with myxoid chondrosarcoma, i.e., predominantly (but not universally) in tumors classically associated with anti-Hu antibodies. In contrast, the cases involving anti-Ma2 antibodies have all occurred in patients with tumors not normally associated with anti-Ma2 antibody response, including renal cell carcinoma (119, 121, 128). In some cases, patients developing paraneoplastic neurological symptoms during immune checkpoint inhibitor therapy had detectable serum titers of paraneoplastic autoantibody *before* checkpoint inhibitor treatment was begun (115, 129, 130). Some—but by no means all—of the reported cases have responded to some extent to treatment with corticosteroids or other modalities, in combination with temporary or indefinite cessation of the specific cancer-directed immunotherapy.

The mechanisms underlying autoimmune and paraneoplastic encephalitides associated with immune checkpoint inhibitors may be disinhibition of an immune response against an autoantigen shared between the tumor and neural tissue and, as noted, some treated patients have had detectable autoantibodies prior to receiving immune checkpoint inhibitors. An experimental model of this type of this disinhibition has recently been reported by Yshii et al. using a genetically modified mouse model that specifically expressed an exogenous neoantigen, hemagglutinin (HA) in Purkinje cells and transplanted tumor cells (96, 131). These mice were then challenged to mount an immune response with a transplanted tumor that also expressed HA. Mice treated with a monoclonal antibody to VLA4 (ipilimumab) to break immune tolerance exhibited neurological disease and Purkinje cell loss. In contrast, in the absence of the anti-VLA4 antibody, none of these mice exhibited any neurological disease or Purkinje cell death. Yshii et al. also detected CD8+ T cells associated with dying Purkinje cells in their animal model and, in addition, reported two human autopsy cases of paraneoplastic cerebellar degeneration in which CD8^+^ T cells were closely associated with areas of Purkinje cell death (96).

## Issues and Implications for Patient Care

Neurological disorders in patients with antibodies to cell surface membrane antigens, such as anti-NMDAR, involve potentially reversible neuronal receptor impairment. Successful treatment of these conditions, with marked patient improvement, has been repeatedly documented following the use of corticosteroids, plasma exchange, intravenous immunoglobulin G (IVIG), and corticosteroid-sparing immunosuppressive agents such as rituximab, cyclophosphamide, or mycophenolate mofetil (132–134). To date, no single agent or sequence of agents has been proven to be more effective, and optimal use of therapeutic agents and length of treatment await prospective controlled clinical trials. The initial response to immunotherapies can be slow, often tempting clinicians to layer multiple immunosuppressive medications without clear endpoints. Recovery is frequently prolonged and requires multidisciplinary support, especially for cognitive and psychiatric symptoms (135).

Neurological injury in patients with paraneoplastic antibodies to *intracellular* neuronal antigens, such as anti-Yo or anti-Hu, represents a different category of disease. Clinical findings in these conditions are ultimately the result of immune-mediated neuronal death, and neurological deficits develop as neurons die and neuronal reserve is exhausted. In these disorders, substantial improvement does not tend to occur, and for this reason, even more than with conditions such as anti-NMDAR encephalitis, time is of the essence in initiating treatment.

Four different considerations come into play in treating affected patients with antibodies such as anti-Yo or anti-Hu. First, early diagnosis and treatment of the underlying tumor, with the removal of the tumor as an antigenic stimulus, is widely considered to provide the greatest chance for neurological improvement or symptom stabilization (136). However, in many cases, this goal cannot be achieved: the underlying tumor may not as yet be detectable or, conversely, the tumor may be sufficiently advanced that treatment is purely palliative. In both instances, immunotherapy may be the mainstay of treatment in the face of as-yet undiagnosed or incurable malignant disease.

The second consideration in treatment has to do timing. In most series and many case reports, treatment was started after symptoms were well-advanced, often weeks or months after symptom onset. Widdess-Walsh et al., in a review of cases of paraneoplastic cerebellar degeneration treated with IVIG, found that most patients having a good response were treated within 1 month of symptom onset, whereas outcome in patients treated after 3 months when neurological deficits were more advanced, was usually limited (137, 138). This finding—that patients with severe deficits respond poorly—has been confirmed by other investigators (139). An important question in many of the reported cases is thus whether treatment was instituted too late to be of value (137–139).

The third consideration has to do with the choice of treatment regimens. Results of treatment reported in the literature have been largely disappointing, with little progress in approaches to treatment over the last 30 years (137–139). Corticosteroids, plasma exchange, immunoadsorption, IVIG, rituximab, cyclophosphamide, and agents such as sirolimus have all been used as immunotherapies (3, 140). However, there has been little uniformity of treatment, even within the given series, and the actual effect of any of these treatments on key T- or B-cell-mediated pathophysiology in paraneoplastic neurological injury remains unclear. Plasma exchange, although effective in reducing serum antibodies, may not sufficiently reduce titers of antibodies produced within the central nervous system (141). Similarly, although rituximab has a profound effect on pre-B cells and B cells, it does not affect plasma cells and in one study did not reduce serum or CSF antibody titers (142). Cyclophosphamide, despite its effects on both T and B cells, may also fail to reduce antibody titers in paraneoplastic neurological disease (143). In the absence of an animal model, the effect of any of these modalities on T-cell function remains undefined. IVIG has multiple potential effects on immune function, but we do not yet know which of these is important in preventing the progression of paraneoplastic neurological injury. Although the use of the plasma cell depleting agent, Bortezomib, has not been reported in paraneoplastic disorders associated with antibodies to *intracellular* antigens, the agent has been shown to reduce antibody titers and produce clinical improvement in NMDAR encephalitis (144).

An additional consideration has to do with the potential role of antineuronal antibodies in causing paraneoplastic neurological disease. Tissue culture studies demonstrating uptake of antibody by neurons raise the question as to whether the use of agents capable of blocking neuronal antibody uptake might represent a potential adjunctive therapeutic approach to these disorders, providing some degree of protection of neurons from antibodies already present in CSF and brain. Colchicine has been shown to prevent uptake of anti-Yo antibodies in slice cultures, presumably by its effect on microtubules (111); however, its clinical use would be limited by its toxicity and narrow therapeutic range. Congdon et al., in studies of a mouse model of Alzheimer's disease, demonstrated that neuronal antibody uptake could be blocked by the clathrin inhibitor, chlorpromazine (145). In the case of paraneoplastic cerebellar degeneration associated with anti-Yo antibody, work by Panja et al. suggests that minimizing intracellular calcium overload toxicity either directly with cyclosporin-A or indirectly with cannabidiol or the ROS scavenger butylated hydroxytoluene could potentially provide neuroprotection by stabilizing mitochondrial calcium homeostasis (110, 113).

A final concern has to do with the way forward in treating this group of patients. The rarity of the classical paraneoplastic disorders and the small number of cases seen at any one institution make conventional multi-institutional controlled trials difficult to achieve. An alternative approach, facilitated by the rapid growth of autoimmune neurology as a specialty, could be a study involving a large number of sites in which enrollment required patients to be ambulatory and cognitively intact, and in which specific, uniformly applied treatment protocols are used. Ultimately, understanding of pathogenesis and imaginative development of therapeutic approaches to this group of disorders awaits the development of successful animal model systems in which such treatments could be tested, and their effects on T cell function and on antibody uptake by neurons and neuronal injury could be studied in detail.

## Author Contributions

JG, NC, SC, and CV conceived and wrote the initial draft of this manuscript. JA and IH contributed to the manuscript draft. JG, NC, SC, JA, IH, and CV completed the final revision as submitted. All authors contributed to the article and approved the submitted version.

## Funding

This work was supported by Merit Review Awards from the United States Department of Veterans Affairs to (JG and NC), awards from the Western Institute for Biomedical Research to (JG and SC), and by grants from Helse Vest to (CV).

## Conflict of Interest

The authors declare that the research was conducted in the absence of any commercial or financial relationships that could be construed as a potential conflict of interest.

## Publisher's Note

All claims expressed in this article are solely those of the authors and do not necessarily represent those of their affiliated organizations, or those of the publisher, the editors and the reviewers. Any product that may be evaluated in this article, or claim that may be made by its manufacturer, is not guaranteed or endorsed by the publisher.
